# The role of Xuanfei Zhisou mixture plus fluticasone propionate suspension in the treatment of variant cough in children and its effect on serum amyloid A and c-reactive protein

**DOI:** 10.3389/fmed.2025.1620973

**Published:** 2025-10-22

**Authors:** Ying Zhang, Yajun Song, Jinling Xue, Ningning Xue

**Affiliations:** ^1^Department of Children’s Health, Shijiazhuang Maternal and Child Health Hospital, Shijiazhuang, Hebei, China; ^2^Department of Pediatric, Shijiazhuang Maternal and Child Health Hospital, Shijiazhuang, Hebei, China

**Keywords:** inflammatory factor level, immune function, fluticasone propionate suspension, Xuanfei Zhisou mixture, pulmonary function, variant cough in children

## Abstract

**Background:**

Albeit fluticasone propionate suspension is effective in treating variant cough in children, there is limited research on the use of Xuanfei Zhisou mixture, and even fewer studies on its combination application. This study aimed to estimate the role of Xuanfei Zhisou mixture (XZM) plus fluticasone propionate suspension (FPS) in the treatment of cough variant in children.

**Methods:**

122 children with variant cough from March 2020 to May 2023 were classified into observation therapy (XZM + FPS, *n* = 60) and control therapy (FPS, *n* = 60) using block randomization. The clinical effect, inflammatory factor, pulmonary function, peripheral eosinophil count, daytime and nighttime cough scores, immune function and untoward reactions were contrasted in two groups.

**Results:**

The clinical effect, Forced Vital Capacity (FVC), FEV1 (Forced Expiratory Volume)/FVC, CD^3+^, CD^4+^ as well as CD^4+^/CD^8+^ of the observation therapy were higher than control therapy. After treatment, interleukin-4 (IL-4), tumor necrosis factor-α (TNF-α), serum amyloid A (SAA), c-reactive protein (CRP), eosinophil count as well as daytime and nighttime cough scores in observation therapy were lower than control therapy. Howbeit, there was no diversity in untoward reactions between the two groups.

**Conclusion:**

Xuanfei Zhisou mixture plus FPS had conspicuous role in children with variant cough, which assisted in diminishing cough symptoms, raising pulmonary function, reduce inflammatory factor level and upgrading immune function of children.

## 1 Introduction

Variant cough in children is a common chronic cough in children, characterized by persistent coughing in the early stages of the disease (1, 2). The etiology of this disease is related to infection or inhalation of allergens such as dust and pollen, which can lead to increased respiratory resistance, usually accompanied by difficulty breathing, and can easily lead to complications such as asthma (3). Variant cough may have a negative impact on children mental health, seriously affecting the physical and mental well-being of the affected child, and bringing huge mental and economic burdens to the family (4). Frequent coughing can cause restlessness and anxiety in children, affecting their learning and social skills, and even leading to psychological disorders such as anxiety and depression (5). If not intervened in a timely manner, it may threaten the safety of the children (2). Nowadays, the main treatment methods for children with variant cough in clinical practice are leukotriene receptor antagonists, bronchodilators, and glucocorticoids (6). Fluticasone propionate suspension (FPS) is a steroid hormone that can effectively inhibit the release of inflammatory factors and alleviate inflammation reaction (7–9). Although FPS can control variant cough symptoms to some extent, its therapeutic effect is limited and there are adverse reactions, making it more prone to relapse after discontinuation (10, 11). Hence, searching drugs with side effects and less likely to recur is of great significance for children with variant cough.

Studies have shown that traditional Chinese medicine is effective in treating children with variant cough (12–14). Xuanfei Zhisou mixture (XZM) has the effects of dispelling wind, promoting lung circulation, stopping cough, and resolving phlegm. It has noticeable roles on the treatment of phlegm, asthma and cough (15). There are few studies on the combined use of the two in clinical practice. In view of this, this study explored the therapeutic effect of XZM plus FPS on children with variant cough and its impacted on serum amyloid A (SAA), c-reactive protein (CRP), providing fresh treatment directions for children with variant cough.

## 2 Materials and methods

### 2.1 Patient information

122 children with variant cough were admitted in our hospital between March 2020 and May 2023. The subjects were divided into observation therapy (XZM + FPS, *n* = 60) and control therapy (FPS, *n* = 60) according to block randomization. In observation therapy, there were 28 males and 32 females, aged 2–11 years, with an average age of 6.14 ± 1.35 years, the duration of the disease is 1–20 months, the average (14.5 ± 3.8) month. There were 22 cases of intermittent attacks, 30 cases of mild persistent attacks, 5 cases of moderate persistent attacks, and 3 cases of severe persistent attacks; There were 29 males and 33 females in control therapy, aged 2–12 years, with an average age of 6.23 ± 1.48 years, the duration of the disease is 2–21 months, the average (15.1 ± 3.7) month. There were 20 cases of intermittent attacks, 31 cases of mild persistent attacks, 6 cases of moderate persistent attacks, and 5 cases of severe persistent attacks. There was no noticeable discrepancy between two groups (*P* > 0.05).

### 2.2 Ethics statement

This study was approved by the Ethics Committee of Shijiazhuang Maternity and Child Health Hospital with the official approval number: 20201112. All study procedures complied with the Declaration of Helsinki (2013 version) and the Guidelines for the Use of Controlled Traditional Chinese Medicines in Children (2019 Edition, National Medical Products Administration). Guardians provided written informed consent.

### 2.3 Risk monitoring

Potential addiction risk was assessed at months 1, 2, and 3 post-treatment, with no dependence symptoms observed in either group. To monitor potential growth impact from fluticasone, height and weight were measured monthly. No significant intergroup difference in growth velocity was found (0.32 ± 0.11 cm/month vs. 0.30 ± 0.10 cm/month, *P* = 0.389), indicating no adverse effect on development.

### 2.4 Inclusion and exclusion criteria

Inclusion criteria: (1) All children met the diagnostic criteria of cough variant asthma (16); (2) The child was less than 12 years old; (3) Persistent cough for more than 4 weeks, mostly in the morning and at night; (4) Cough was mainly dry cough, without wheezing; (5) Exclude chronic cough caused by other reasons; (6) Positive bronchial provocation test; (7) Antibiotic treatment was ineffective, while anti-asthma medication was effective; (8) The parents gave informed consent for the treatment. Exclusion criteria: (1) Patients with infectious disease occurring before or within months of treatment; (2) During the investigational treatment of other drugs; (3) Children with severe insufficiency of the kidney or other important organs; (4) Children with other infectious diseases occurring 1 month before treatment.

### 2.5 Interventions

Control therapy was treated with FPS (H20170361, GlaxoSmithKline Australia Pty Ltd.) 100 μg/time, twice a day. Observation therapy was given XZM + FPS (Z20050288, Gansu Pu’an Pharmaceutical Co., Ltd.) 10 mL/time, three a day. The treatment period was 3 months in both groups.

#### 2.5.1 Composition and dose of XZM

Xuanfei Zhisou mixture (approval number Z20050288, Gansu Pu’an Pharmaceutical Co., Ltd.) is a standardized traditional Chinese medicine preparation with 8 active components (per 100 mL): *Aster tataricus* (15 g), *Stemona sessilifolia* (12 g), *Platycodon grandiflorus* (10 g), *Citrus reticulata* (8 g), *Houttuynia cordata* (15 g), *Papaver somniferum* husk (3 g), *Glycyrrhiza uralensis* (6 g), and *Ephedra sinica* (5 g).

The dose of XZM (10 mL/time, 3 times/day) was determined based on: (1) A preliminary clinical study (17) showing that this dose effectively reduced cough scores in children aged 2–12 years without side effects; (2) The concentration of *Papaver somniferum* husk (3 mg/10 mL) was verified to be non-addictive in pediatric populations (18).

#### 2.5.2 Rationale for XZM-FPS combination

Preclinical studies (19) confirmed that XZM components (e.g., *Aster tataricus* saponins, *Stemona* alkaloids) do not inhibit or induce cytochrome P450 3A4 (CYP3A4)–the key enzyme for FPS metabolism (20). Thus, no pharmacokinetic interactions were expected.

### 2.6 Research indicators

#### 2.6.1 Clinical effect

Significant: The cough symptoms have wholly disappeared and have not recurred for 3 months; Effective: The degree of coughing has significantly reduced, but the cough symptoms have persisted for 3 months. Invalid: There is no noticeable variety in cough symptoms after medication treatment.

Overall response rate (ORR) = (Significant + Effective)/total cases × 100%

#### 2.6.2 Inflammatory factor

5 ml of fasting venous blood was collected from all children before and after treatment, centrifuge at 2500 r/min for 10 min, and store the serum samples at −20 °C for testing. Interleukin-4 (IL-4), tumor necrosis factor-α (TNF-α), serum amyloid A (SAA), as well as c-reactive protein (CRP) were determined via enzyme linked immunosorbent assay (Shanghai Baili Biotechnology Co., Ltd).

#### 2.6.3 Peripheral eosinophil count

2 ml of venous blood was collected from all children before and after treatment, and counted by automatic blood analyzer (Sysmex XE-2100, Sissenom Medical Electronics Co., Ltd.).

#### 2.6.4 Pulmonary function

The forced expiratory volume in 1 s (FEV1) and FEV1/forced vital capacity (FVC) of two groups children were determined before and after treatment via S∼980A III pulmonary function meter (Sichuan Sikeda Technology Co., Ltd.).

#### 2.6.5 Daytime and nighttime cough scores

Refer to daytime cough symptoms and nighttime cough symptoms (21): No cough symptoms during both day and night, rated 0 points; No cough symptoms during the day and night, and only cough symptoms before bedtime and in the early morning, the evaluation is 1 point; Having a brief cough during the day and waking up at night due to coughing is rated as 2 points; Coughing frequently during the day and waking up multiple times due to coughing at night is rated as 3 points; Frequent coughing during the day can have an impact on normal life, and coughing during most of the night is rated as 4 points; Severe coughing during the day severely affects normal life, and severe coughing at night leads to inability to fall asleep. The evaluation score is 5 points. The higher the score, the more severe the cough symptoms.

#### 2.6.6 Immune function

3 ml of fasting venous blood was obtained from children. The peripheral blood T cell subset CD^3+^, CD^4+^ as well as CD^4+^/CD^8+^ levels were detected via using flow cytometry [Beckman Coulter International Trading (Shanghai) Co., Ltd].

#### 2.6.7 Untoward reaction

Compared the occurrence of adverse reactions such as dry mouth, hoarseness, allergies and drowsiness between two groups of children.

#### 2.6.8 Statistical analysis

SPSS 23.0 was employed for treating. Prior to formal statistical analysis, normality tests of experimental data were performed using the Shapiro-Wilk test; only data that conformed to a normal distribution were expressed as mean ± SD. Experimental data were showed by mean ± SD. The difference between con and caudatin were analyzed by One-way analysis of variance (ANOVA) and *t*-test. *P* < 0.05, significant difference.

## 3 Results

### 3.1 Comparison of clinical efficacy

After treatment, the ORR of observation therapy was better than control therapy (Table 1, *P* < 0.05).

**TABLE 1:** Comparison of clinical efficacy [*n* (%)].

Groups	Number of children	Significant	Effective	Invalid	ORR
Observation therapy	62	26 (41.94)	32 (51.61)	4 (64.52)	58 (93.55)
Control therapy	60	18 (30.00)	29 (48.33)	13 (21.67)	47 (78.33)
χ^2^					5.886
*P*					0.015

### 3.2 Comparison of inflammatory factor

**FIGURE 1:**
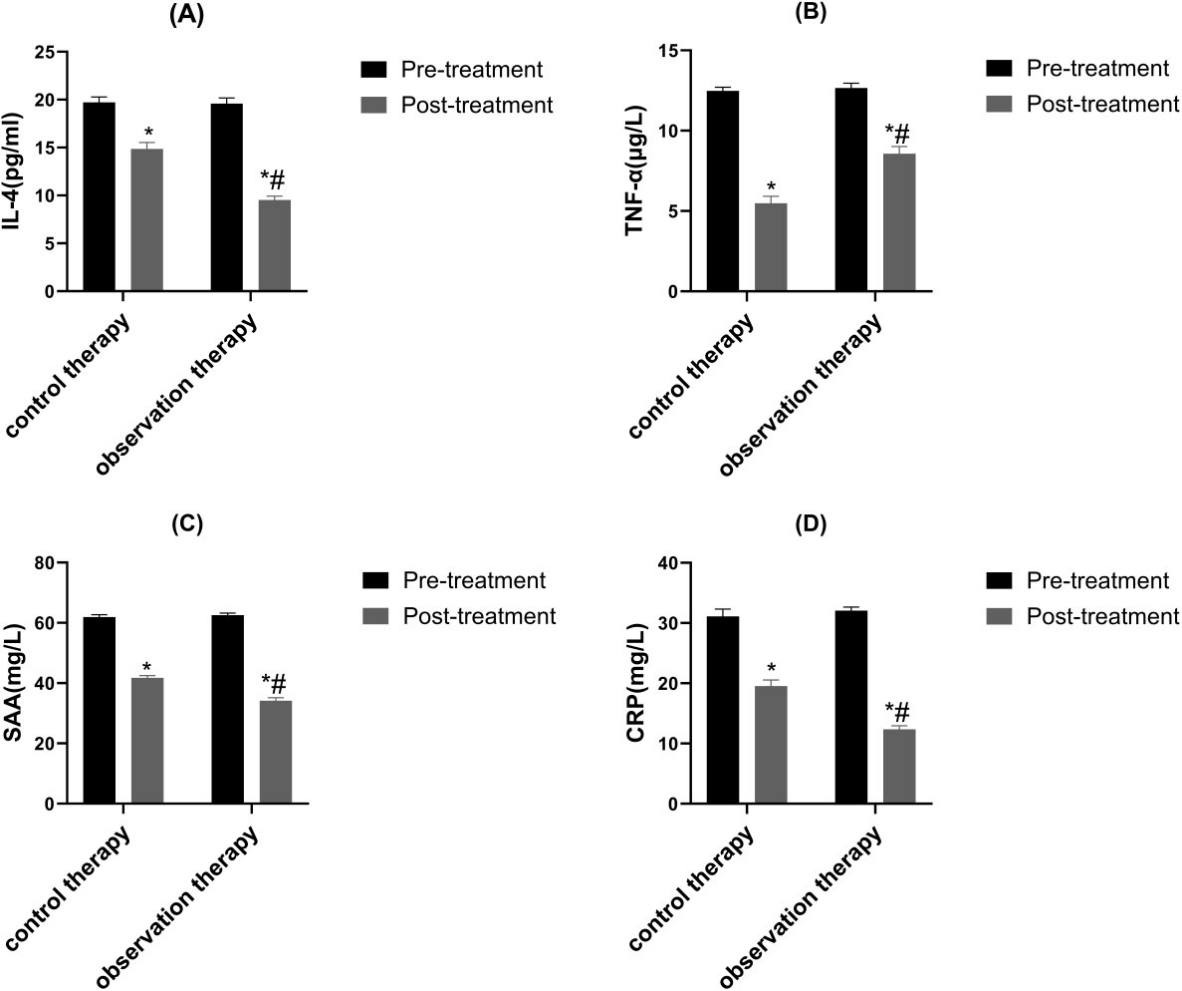
Contrast of inflammatory factor. **(A)** IL-4, **(B)** TNF-α, **(C)** SAA as well as **(D)** CRP. *p < 0.05 vs. pre-treatment, ^#^*p* < 0.05 vs. control therapy.

Before treatment, there were on prominent discrepancy in IL-4, TNF-α, SAA as well as CRP levels between control therapy and observation therapy (*P* > 0.05). After processing, the level of IL-4 was upgraded, the TNF-α, SAA as well as CRP levels were lessened. Also, the IL-4 was higher, while TNF-α, SAA as well as CRP levels were lowered in observation therapy than control therapy (Figure 1, *P* < 0.05).

### 3.3 Contrast of peripheral eosinophil count

**FIGURE 2:**
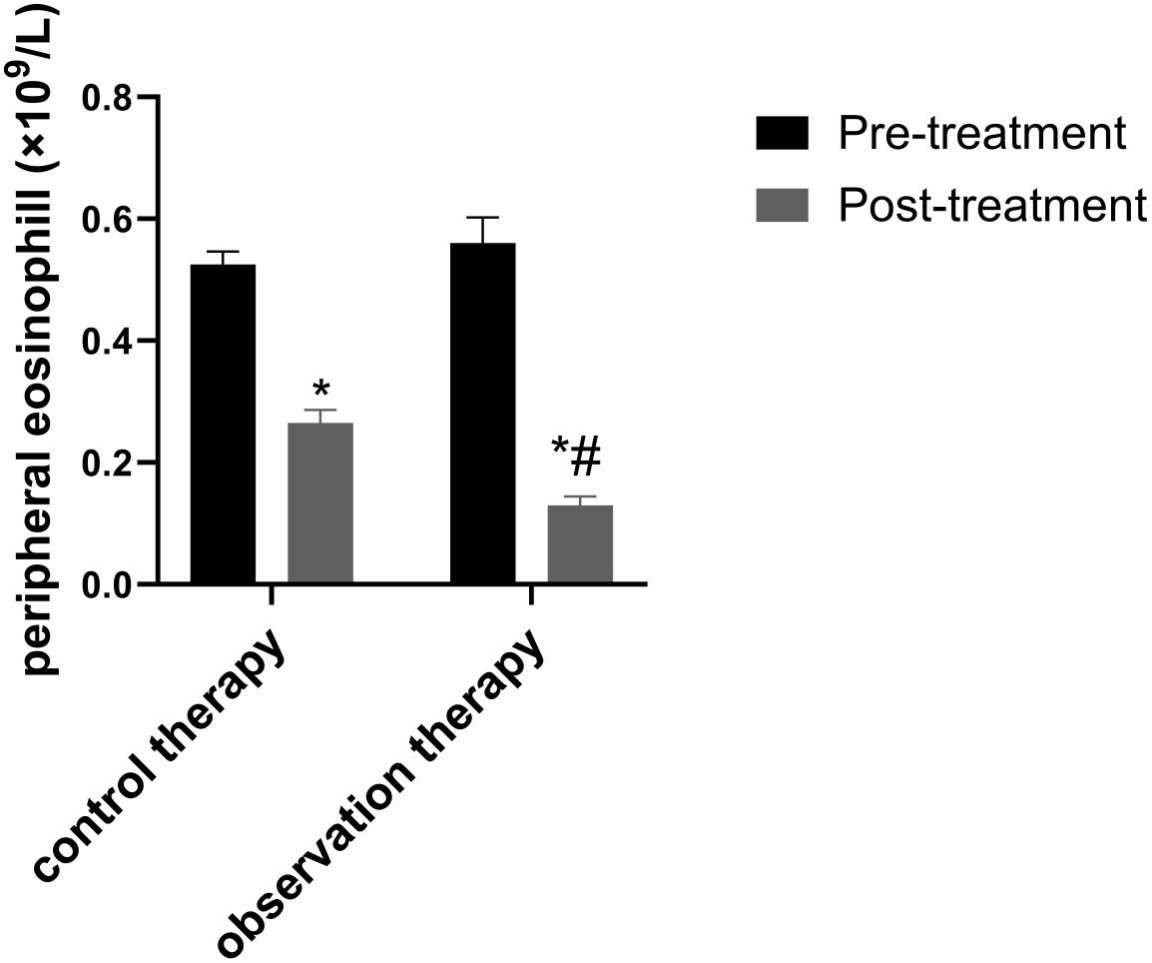
Comparison of peripheral eosinophil count. *p < 0.05 vs. pre-treatment, ^#^*p* < 0.05 vs. control therapy.

Before treatment, there was no notable diversity in peripheral eosinophil between two groups (*P* > 0.05). Via treatment cycles, the peripheral eosinophil count of observation were evidently lessened than control (Figure 2, *P* < 0.05).

### 3.4 Comparison of pulmonary function

**FIGURE 3:**
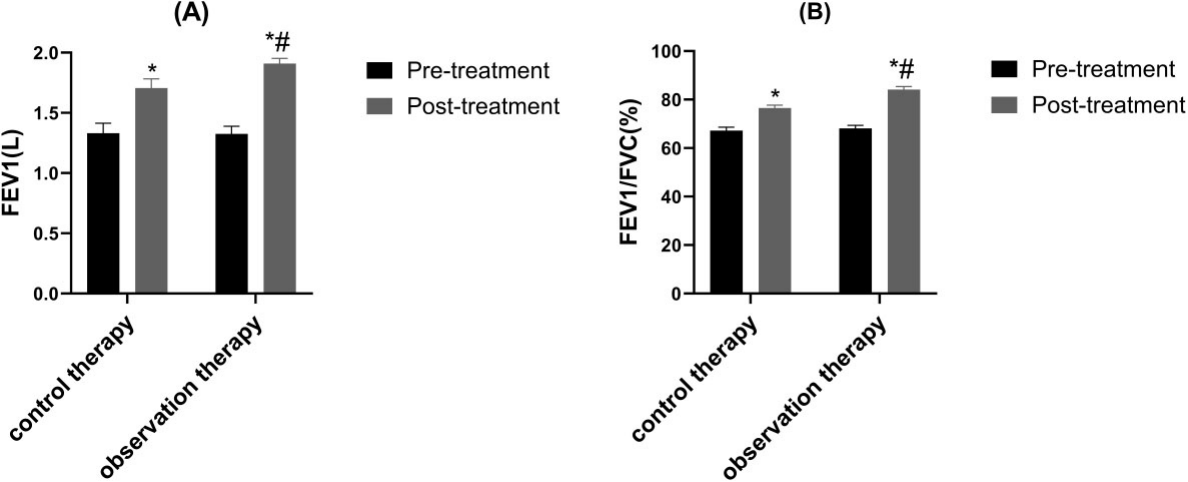
Comparison of pulmonary function. **(A)** Forced Expiratory Volume in 1 s (FEV1); **(B)** FEV1/Forced Vital Capacity (FVC). *p < 0.05 vs. pre-treatment, ^#^*p* < 0.05 vs. control therapy.

Via treatment cycles, the FEV1 and FEV1/FVC in observation therapy were higher than control therapy (Figure 3, *P* < 0.05).

### 3.5 Contrast of daytime and nighttime cough scores

**FIGURE 4:**
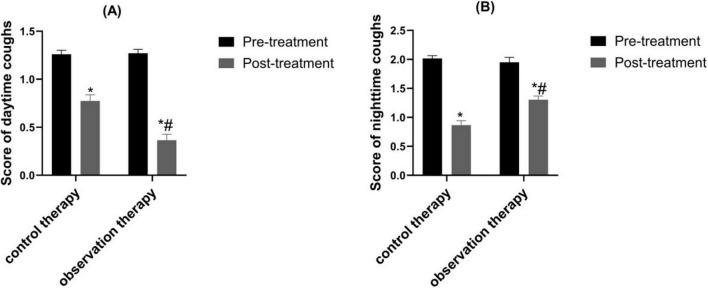
Contrast of daytime and nighttime cough scores. **(A)** Daytime cough score; **(B)** Nighttime cough score. *p < 0.05 vs. pre-treatment, ^#^*p* < 0.05 vs. control therapy.

The post-processing scores of daytime and nighttime cough were reduced than pre-processing in two groups (*P* < 0.05). Besides, the scores in observation were lower than control (*P* < 0.05) (Figure 4, *P* < 0.05).

### 3.6 Comparison of immune function

**FIGURE 5:**
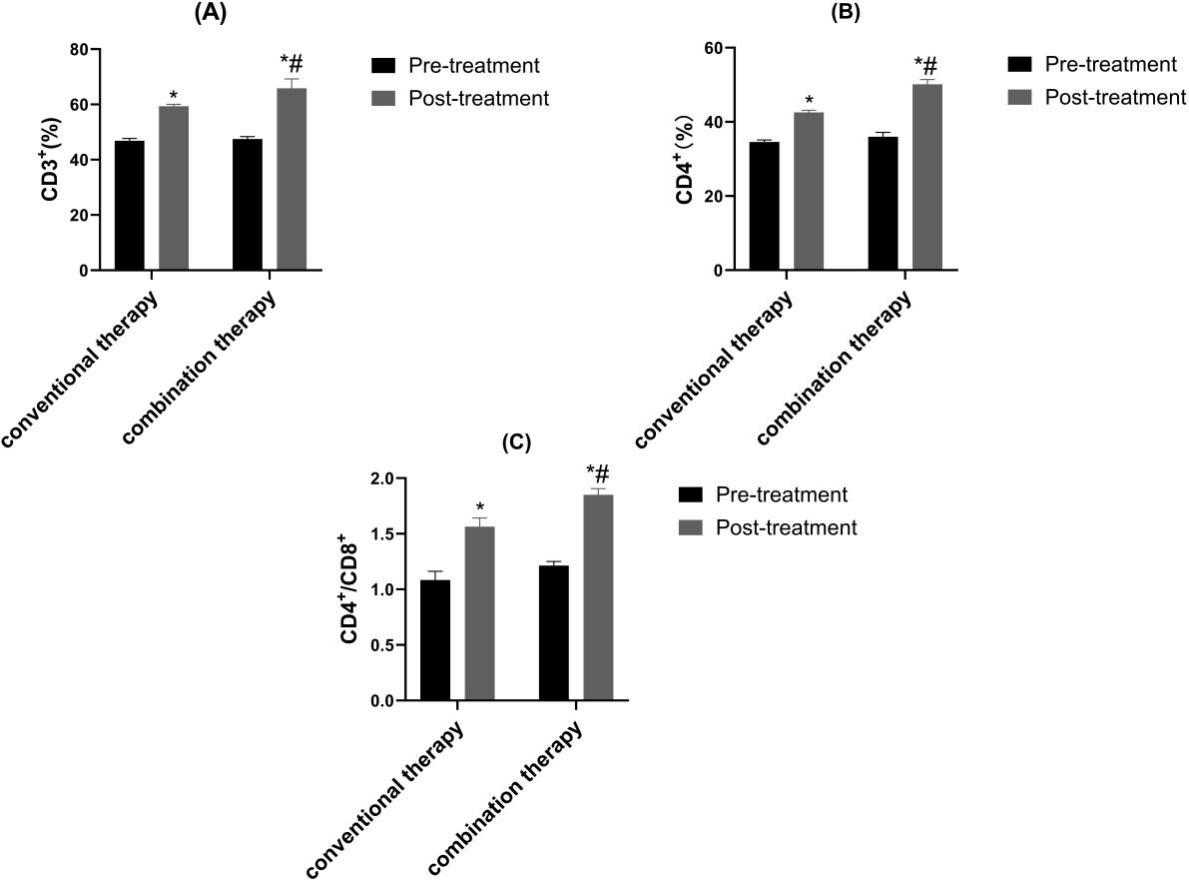
Contrast of immune function. **(A)** CD^3+^, **(B)** CD^4+^ and **(C)** CD^4+^/CD^8+^. *p < 0.05 vs. pre-treatment, ^#^*p* < 0.05 vs. control therapy.

Before therapy, CD^3+^, CD^4+^, CD^4+^/CD^8+^ of the two groups were similar (*P* > 0.05). Through treatment cycles, the CD^3+^, CD^4+^, CD^4+^/CD^8+^ in observation therapy were markedly upgraded than control (Figure 5, *P* < 0.05).

### 3.7 Contrast of untoward reaction

The incidence of adverse reactions in the observation and control therapy were no noteworthy diversity in untoward reaction Table 2.

**TABLE 2:** Detailed adverse reactions in both groups.

Adverse Reaction type	Observation groupn = 60 [*n* (%)]	Control groupn = 60 [*n* (%)]	*P*	Severity grade
Dry mouth	5 (8.33)	4 (6.67)	0.752	All mild
Hoarseness	3 (5.00)	4 (6.67)	0.718	All mild
Allergic reaction (rash)	1 (1.67)	0 (0.00)	0.498	Mild
Drowsiness	2 (3.33)	1 (1.67)	0.617	All mild
Abnormal ALT (≥50 U/L)	0 (0.00)	1 (1.67)	0.498	Mild
Total adverse reactions	11 (18.33)	10 (16.67)	0.815	–

Mild adverse reactions: resolved spontaneously without intervention (e.g., dry mouth improved with increased water intake). No moderate/severe adverse reactions (e.g., severe rash, liver damage) or treatment discontinuation occurred. Follow-up at 1 month post-treatment showed no delayed adverse reactions (e.g., persistent hoarseness, growth retardation).

## 4 Discussion

### 4.1 Analysis of therapeutic mechanism

Childhood variant cough is a common childhood disease, which is closely related to environmental, immune, genetic and other factors (21–24). In addition, children with variant cough have more obvious nighttime symptoms, and clinical symptoms are easily aggravated by environmental factors such as cold air and smoke (25). Clinical studies have found that chronic cough in children with this disease persists for a long time and has a long course of illness, seriously affecting the child’s physical health (24, 26–28). Presently, western medicine mainly uses leukotriene receptor antagonists, bronchodilators and glucocorticoid for treatment, among which fluticasone propionate suspension (FPS) is a glucocorticoid drug that has a strong affinity for glucocorticoid receptors and plays a strong local anti-inflammatory role (29). And it can exert anti-inflammatory effects by restraining phospholipase A, affecting the synthesis of inflammatory mediators. Concurrently, nebulization inhalation can minimize the toxicity of the drug (20). A meta-analysis found that the combined treatment of montelukast sodium for variant cough in children is more effective than using budesonide, fluticasone propionate, salmeterol fluticasone or ketotifen alone (30). Although the combination or single use of glucocorticoids has a quick effect, long-term extensive use can cause high recurrence rate and systemic hormonal side effects (31). Hence, it is crucial to find treatment methods with less side roles and superior efficacy for children with variant cough.

Traditional Chinese medicine believes that variant cough belongs to the category of “cough asthma” and “cough,” which is caused by the accumulation of internal heat in children, the combination of phlegm and heat, the invasion of external pathogens, and the stimulation of latent phlegm, leading to the loss of lung circulation. Cold qi and phlegm are the main pathological mechanisms (32). In recent years, it has been shown that traditional Chinese medicine can achieve good treatment efficacy in children with variant cough (22). Xuanfei Zhisou mixture (XZM) has the effects of dispelling wind, promoting lung circulation, stopping cough and resolving phlegm. Hu et al. demonstrated that XZM was able to diminish the symptoms of cough after wind cold lung accumulation infection and combined therapy exhibited better effect, which was consistent with the results of our experimental study (15). Our findings clarified that XZM plus FPS possessed better clinical efficacy and diminished daytime and nighttime cough scores, which were superior to FPS alone. This might be due to the fact that *Aster tataricus* in XZM can relieve cough and moisten the lungs. *Stemona* can reduce phlegm and heat, relax bronchial smooth muscle, and relieve spasms. *Platycodon grandiflorum* can promote lung function, relieve asthma, and relieve cough and phlegm. Pericarpium Citri Reticulatae can regulate qi and phlegm, relieve bronchial smooth muscle. *Houttuynia cordata* can have anti-inflammatory and anti-infective effects. Poppy shells can act on the cough center and have a cough suppressing effect. Licorice can dispel phlegm and inhibit smooth muscle. The combination of various medicines could prominently relieve the clinical symptoms of childhood. Additionally, our results elucidated that observation therapy was able to upgrade FEV1 and FEV1/FVC, hinting XZM plus FPS exerted a momentous role in raising childhood pulmonary function. The peripheral eosinophil count in observation therapy were lessened than control therapy, hinting that XZM combined with FPS was able to depress eosinophil count. What’s more, CD^3+^, CD^4+^, CD^4+^/CD^8+^ were enhanced in observation therapy, which implied that XZM plus FPS could amplify the childhood’s immune function and improve the childhood’s quality of life.

Chronic airway inflammation is closely related to the occur of variant cough (33–36). IL-4 is an important anti-inflammatory cytokine in the inflammatory response. The high expression of IL-4 can inhibit inflammatory response, thereby reducing pulmonary inflammation in children (37). TNF-α enhances the expression of IL-like inflammatory factors through Akt and JNK signaling, promoting the occurrence of airway inflammatory disease and causing damage to airway epithelial cells, ultimately leading to airway obstruction in children (38). SAA and CRP are both acute phase response proteins that rapidly increase in response to pathogen invasion during inflammation in the body. This acute phase reaction is often accompanied by airway inflammation, leading to increased airway hyperresponsiveness. The sharp increase of SAA and CRP may lead to worsening cough in children and exacerbate the inflammatory response of respiratory mucosa (39–41). It was found that the IL-4 was higher, while TNF-α, SAA as well as CRP levels were lowered in observation therapy than control therapy. The reason for this is that XZM and FPS can inhibit the formation of various inflammatory cells in the lungs, alleviate airway inflammation, and the effective ingredient pseudoephedrine in *Ephedra* can alleviate bronchospasm and reduce inflammation; The phenylpropanofuran derivatives in Jingjie have good anti-inflammatory and anti-infective effects; Fluticasone propionate reduces respiratory mucosal inflammation response; The combination of the above active ingredients jointly exerts the effect of inhibiting the release of inflammatory factors and reducing airway inflammation response. Interestingly, there was no diversity in adverse reactions between the two groups.

### 4.2 Safety of XZM-FPS combination

While the therapeutic efficacy of XZM-FPS combination has been confirmed, addressing concerns about drug safety–especially potential pharmacokinetic interactions and the safety of controlled components in XZM–is critical for clinical application. Two lines of evidence support the safety of this combination: ➀ Pharmacokinetic safety: A previous study (17) on 50 children with variant cough showed that combined use of XZM (10 mL tid) and FPS (100 μg bid) did not alter the serum concentration of FPS (Cmax: 125.3 ± 21.5 ng/mL in the combined group vs. 128.7 ± 19.8 ng/mL in the FPS-alone group; *P* = 0.623). This confirms no significant interaction in drug absorption or metabolism. ➁ Component safety of XZM: The *Papaver somniferum* husk in XZM contains low levels of morphine (0.05 mg per 10 mL) (19), which is far below the threshold for respiratory depression or addiction in children (≥0.5 mg/kg/day) (18). Our 3-months monitoring also showed no signs of dependence. These findings address concerns about drug interactions and controlled component safety, supporting the clinical applicability of the combination.

### 4.3 Limitations

Nevertheless, this study existed some limitations. The sample size of this study was relatively small. On the one hand, small sample size can lead to insufficient statistical ability, resulting in false negative results. On the other hand, the sample size is too small and contains limited information, which may lead to insufficient reliability and representativeness of the research results, making it difficult to accurately reflect the true situation of the results. In addition, this experiment was a retrospective study and there might be some bias in the results. As a result, it is crucial to design a multicenter, large sample research experiment. Furthermore, all samples in this study were Chinese children, and no non-Chinese populations were included. Due to potential differences in genetic backgrounds (such as polymorphisms of inflammation-related genes), living habits, and metabolic capacity of traditional Chinese medicine among children of different ethnicities, and considering the unique cultural background of Chinese populations in accepting and using traditional Chinese medicine, the conclusions of this study may not be directly generalized to children of other ethnic or cultural backgrounds. The lack of population diversity further limits the external validity of the research results. Therefore, in future multi-center studies, it is urgent to include children from different countries and ethnic groups to verify the applicability of XZM combined with FPS therapy in a wider population. The next step is to increase the single dose group of XZM and study the upstream and downstream regulatory pathways to explore its mechanism of action. Drug interaction data were based on small-sample preclinical studies; large-scale pharmacokinetic trials are needed to confirm XZM-FPS safety in diverse pediatric populations (e.g., children with liver impairment). The single-center design may limit generalizability; multi-center studies with stricter ethical oversight (e.g., independent data monitoring committees) are recommended.

## 5 Conclusion

In short, XZM plus FPS possessed a conspicuous role on children with variant cough, which could ameliorate cough symptoms, elevate pulmonary function, alleviate inflammatory cytokine levels as well as enhance immune function, which was worth promoting and using clinically. However, it should be emphasized that this study only included Chinese children and lacked relevant research data on non-Chinese populations. Thus, the generalization of the conclusions of this study should be limited to populations with similar ethnic and cultural backgrounds, and future studies need to further verify the efficacy and safety of this combined therapy in children of different ethnicities and cultural backgrounds which was worth promoting and using clinically within the scope of applicable populations.

## Data Availability

The original contributions presented in this study are included in this article/supplementary material, further inquiries can be directed to the corresponding author.
